# Effect of body mass index on *in vitro* fertilization outcomes in women

**DOI:** 10.4103/0974-1208.74155

**Published:** 2010

**Authors:** Anjali Sathya, Sathya Balasubramanyam, Shalu Gupta, Thankam Verma

**Affiliations:** Consultant Endocrinologist, Institute of Reproductive Medicine and Women’s Health, Madras Medical Mission Hospital, Chennai, Tamil Nadu, India; 1 Consultants in Reproductive Medicine, Institute of Reproductive Medicine and Women’s Health, Madras Medical Mission Hospital, Chennai, Tamil Nadu, India

**Keywords:** Obesity, infertility, pregnancy outcome

## Abstract

**BACKGROUND:**

Obesity has become a major health problem across the world. In women, it is known to cause anovulation, subfecundity, increased risk of fetal anomalies and miscarriage rates. However, in women going for assisted reproduction the effects of obesity on egg quality, embryo quality, clinical pregnancy, live birth rates are controversial.

**OBJECTIVES:**

To assess the effect of women’s body mass index (BMI) on the reproductive outcome of non donor In vitro fertilization (IVF)/Intracytoplasmic sperm injection (ICSI). The effects of BMI on their gonadotrophin levels (day 2 LH, FSH), gonadotrophin dose required for ovarian stimulation, endometrial thickness and oocyte/embryo quality were looked at, after correcting for age and poor ovarian reserve.

**MATERIALS AND METHODS:**

Retrospective study of medical records of 308 women undergoing non donor IVF cycles in a University affiliated teaching hospital. They were classified into three groups: normal weight (BMI<25 kg/m^2^), overweight (BMI>25 <30 kg/m^2^) and obese (BMI>30 kg/m^2^). All women underwent controlled ovarian hyper stimulation using long agonist protocol.

**RESULTS:**

There were 88 (28.6%) in the normal weight group, 147 (47.7%) in the overweight and 73 (23.7%) in the obese group. All three groups were comparable with respect to age, duration of infertility, female and male causes of infertility. The three groups were similar with respect to day 2 LH/FSH levels, endometrial thickness and gonadotrophin requirements, oocyte quality, fertilization, cleavage rates, number of good quality embryos and clinical pregnancy rates.

**CONCLUSION:**

Increase in body mass index in women does not appear to have an adverse effect on IVF outcome. However, preconceptual counselling for obese women is a must as weight reduction helps in reducing pregnancy-related complications.

## INTRODUCTION

Obesity is becoming a fast growing health problem across the world. In addition to diabetes and cardiovascular disease, it also leads to alterations in reproductive functions. Excess body fat leads to menstrual irregularities especially chronic oligo-anovulation and infertility. Obesity leads to hyperinsulinemia and consequent ovarian hyperandrogenism.

Obesity is also known to affect fertility. Several prospective studies have shown that obesity leads to anovulatory infertility. It is associated with higher miscarriage rates and higher prevalence of gestatational diabetes and pregnancy induced hypertension.[1–3]

In assisted reproduction, however, there are conflicting reports on the effect of obesity on oocyte quality, embryo development, lower number of mature oocytes, lower implantation and pregnancy rates.[4–8] Endometrium also seems to have a negative impact on reproductive outcome in studies based on oocyte donation model. The objective of our study is to assess the effect of women’s BMI on the oocyte/embryo quality and the reproductive outcome of our non donor IVF/ICSI cycles.

## MATERIALS AND METHODS

We conducted a retrospective analysis of the case records of 308 consecutive women who underwent non donor IVF cycles in our institute. They were classified into three groups based on their BMI i.e. normal weight (BMI<25 kg/m^2^), overweight (25-30 kg/m^2^ and obese (>30 kg/m^2^). Potential confounding factors like the woman’s age, origin of infertility and ovarian reserve were noted. Those women older than 40 years and with FSH more than 10mIU/ml were excluded from the analysis.

All women underwent controlled ovarian hyperstimulation using long agonist protocol. This involved initial down regulation with GnRH agonist from the mid luteal phase of the previous menstrual cycle and maintained till human chorionic gonadotrophin (hCG) administration. Recombinant FSH ± HMG was started from day 2/3 of next cycle and the dosage was decided based on the patient’s age, antral follicle count, basal FSH/LH levels and response to previous ovarian stimulation. Their dosages were adjusted according to serum LH, estradiol levels and ovarian response. hCG was administered subcutaneously when at least 3 follicles were ≥ 18 mm in dimension. Oocyte retrieval was done 36 h later followed by embryo transfer on day 3.

Oocytes with expanded cumulus, radiant corona, distinct zona pellucida, clear cytoplasm, unfragmented first polar body and those without debris in the perivitelline space were considered as good.[9] After oocyte retrieval, fertilization was assessed the next day (day 1) and embryo cleavage 24 h later. Embryo morphology was studied on day 3 based onthe number of cells and fragmentation using the standard morphological criteria. A good day 3 embryo was defined as one with number of blastomeres greater than or equal to 8, fragmentation of < 20% and without multinucleated blastomeres.[10]

Pearson Chi-square tests were used to compare the binary variables between the various groups classified according to the BMI. Parametric tests were used for comparisons between the BMI groups and continuous variables. Analysis of variance (ANOVA) was then performed and post-hoc comparisons were made.

## RESULTS

**Figure 1:**
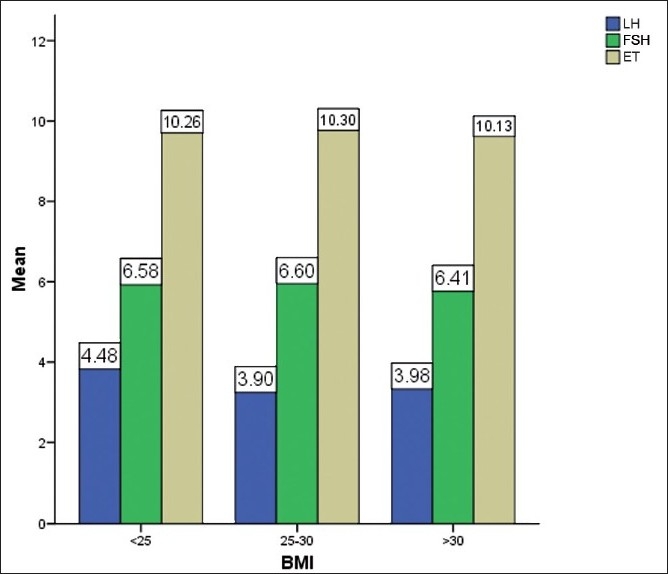
Effect of BMI on LH, FSH, endometrial thickness and gonadotrophin dose

**Figure 2:**
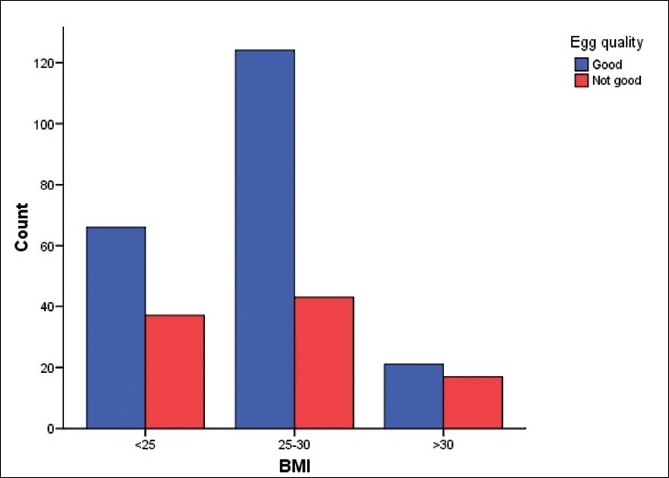
Effect of BMI on oocyte quality <25–48 good, 24 not good 25-30-90 good, 26 not good, >30–16 good, 10 not good *P* = 0.12

**Figure 3:**
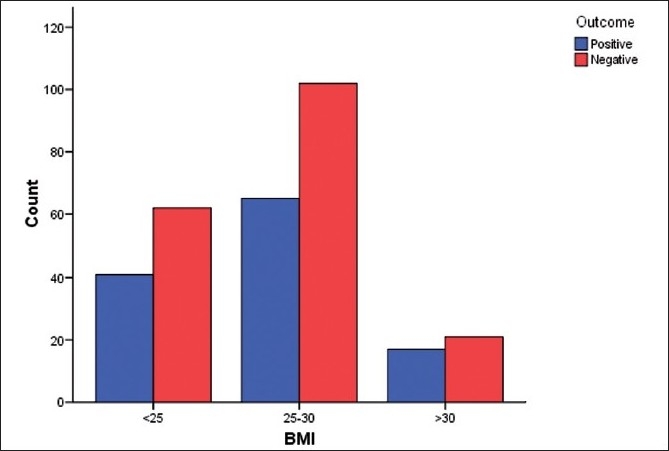
Effect of BMI on pregnancy outcome <25 – 34 +, 38-, (total = 72), 25-30 – 52+, 64-, (total = 116), >30– 12+, 14–, (total = 26), P-0.95 (not significant)

There was a total of 308 women. There were 88 (28.6%) in the normal weight group, 147 (47.7%) in the overweight and 73 (23.7%) in the obese group. 3 among the obese were morbidly obese. All three groups were comparable with respect of infertility [Table 1]. There was no significant difference in the day 2 gonadotrophin values (FSH, LH), endometrial thickness and gonadotrophin dose required for controlled ovarian stimulation [Table 2, Figure 1]. The oocyte quality did not vary between the three groups [Figure 2]. The correlation studies between the body mass indices and the pregnancy rates, implantation rates and fertilization rates failed to show any significant association [Figure 3]. The number of missed abortions and ectopic pregnancies were comparable in the three groups [Table 3].

**Table 1:** Baseline characteristics

BMI	<25kg/m^2^	25-30 kg/m^2^	>30kg/m^2^	*P*
No.	88	147	73	
Age (Mean±SD)	30.3±3.2	31.4±2.6	33.3±3.9	0.82
BMI (Mean±SD)	22.5±2.4	27.2±2.2	32.1±3.7	
Duration of infertility	7.5±1	6.8±0.6	8.8±1.03	0.33
E2 on hCG day	2113±240	1701±141	1403±165	0.68
Women with PCOS (%)	14.7%	12%	10.2%	0.12
Male fact (%)	50	53	61	0.39
Duration of COH(d)	11.2	11.02	10.6	0.78
Good oocyte quality (%)	64.7%	75.5%	64.4%	0.28
No. of good embryos transferred	3.3 ± 0.8	3.6 ± 1.1	3.5 ± 0.7	0.63

BMI: Body mass index, PCOS: Polycystic ovarian syndrome, COH: Controlled ovarian stimulation

**Table 2:** Day 2 LH/FSH levels, endometrial thickness and gonadotrophin requirements in the three groups

BMI	<25kg/m^2^ Mean±SD	25-30 kg/m^2^ Mean±SD	>30kg/m^2^ Mean±SD
LH 95% CI ANOVA (0.09)	4.36 ± 2.48 [3.78-4.95]	3.74 ± 2.15 [3.34-4.14]	3.38 ± 2.31 [2.45-4.31]
FSH ANOVA (0.86)	5.49 ± 1.26 [5.19-5.79]	5.49 ± 1.17 [5.27-5.71]	5.36 ± 1.03 [4.94-5.78]
ET ANOVA (0.32)	10.12 ± 1.23 [9.8-10.5]	10.47 ± 1.48 [10.1-10.7]	10.23 ± 1.48 [9.5-10.5]
Gonadotrophin dose ANOVA (0.42)	2490.6 ±1057.6 [2242-2739]	2690.7 ±1048 [2497-2884]	2658 ±887.3 [2292-3025]

ANOVA: Analysis of variance, BMI: Body mass index

**Table 3:** Outcomes in the 3 BMI groups

BMI	<25kg/m^2^	25-30 kg/m^2^	>30kg/m^2^	*P*
Clinpreg. positive	38 (43)	66 (44.8)	24 (32.8)	0.95
Missed abort.	2	12	8	0.62
Multiple preg.	10 (11)	16 (10.8)	13 (17)	0.28
ectopic	1(1.1)	3 (2)	3 (4)	0.66
Implant. rate	(19.3)	(21.4)	(20.56)	0.38

BMI: Body mass index, Figures in parentheses are in percentage

## DISCUSSION

Anovulation has been the major cause of subfertility in obese women.[1] Reduced conception rates (5% per unit increase in BMI above 29 kg/m^2^) have been described in normal cycling obese women as well. The effects of BMI in obese women undergoing controlled ovarian stimulation have been controversial.[1,4–6]

Our study shows that BMI has no adverse effects over IVF outcome. The blood hormone parameters (day 2 gonadotrophin values, estradiol levels on hCG day) were similar in all three groups irrespective of weight. Obese women are expected to have high LH values owing to insulin resistance. However, we did not find the same in our study. Some studies have highlighted a state of gonadotrophin resistance in obese women leading to higher gonadotrophin requirement for COH.[4] Some studies have not found any adverse effects of obesity on ovarian response in IVF. Our study groups comprising overweight and obese women showed similar gonadotrophin requirements as that of normal weight women. Also, the number of days of controlled ovarian hyperstimulation was similar in all the three groups.

With regards to oocyte and embryo quality, some authors have reported poor quality among obese women.[8] Others have failed to find any association between the two.

Lacunae still exists as regards the role of endometrium, the embryo in implantation in obese women. Many studies indicate lower implantation and pregnancy rates, higher miscarriage rates and increase pregnancy- related complications for the mother and the fetus.[11] Nichols *et al*., report reduced conception rates in overweight women undergoing IVF.[2] Wang *et al*., also support the same. However, there have been conflicting conclusions from other studies.[3]

High miscarriage rates have been described in obese women conceiving naturally or after ART. A systematic review by Maheshwari *et al*., made similar observations in women with BMI greater than 25 kg/m^2^.[4] Metwally *et al*., in his meta analysis also observe similar trend in BMI greater than 25 kg/m ^2^ regardless of method of conception[8] In our study, we did not observe any difference between the three groups. This could be explained by the fact that the embryo quality was not affected by BMI.

Our study thus does not show any adverse effects of obesity on endometrial thickness, hormone levels, oocyte number and quality, implantation and pregnancy rates. The role of endometrium and factors related to embryo are still under study. Other confounding factors like the role of male obesity on semen quality have not been taken into account. We need to study the crosstalk between the endometrium and the embryo at molecular level and study obesity in male partner, the effect of obesity on semen quality and hence embryo quality.

We conclude that an increase in body mass index does not appear to have an adverse effect on IVF outcome. However, overweight and obese women must be counselled on weight reduction to reduce pregnancy-related complications.
